# Iron deficiency, fatigue and muscle strength and function in older hospitalized patients

**DOI:** 10.1038/s41430-020-00742-z

**Published:** 2020-09-08

**Authors:** Sophia Neidlein, Rainer Wirth, Maryam Pourhassan

**Affiliations:** Department of Geriatric Medicine, Marien Hospital Herne, Ruhr-Universität Bochum, Herne, Germany

**Keywords:** Risk factors, Nutrition disorders, Fatigue

## Abstract

**Background/Objectives:**

Iron deficiency is common in older patients. We investigated whether iron deficiency is an independent risk factor for functional impairment, low muscle function, fatigue, and rehabilitation progress in older hospitalized patients.

**Subjects/Methods:**

Two hundred twenty-four patients (age range 65–95 years; 67% females) who were consecutively admitted to a geriatric acute care ward participated in this prospective longitudinal observational study. Ferritin, iron, transferrin in serum, and blood hemoglobin were measured and current iron supplementation was recorded. Fatigue and comorbidity were measured using the fatigue severity scale and Charlson Comorbidity Index, respectively. Barthel Index, handgrip strength, and isometric knee extension strength were conducted at the time of hospital admission and before discharge.

**Results:**

Ninety-one (41%) patients had iron deficiency in which the majority had functional iron deficiency (78/91, 86%). Absolute iron deficiency with and without anemia was diagnosed in 12 (13%) and one patients, respectively. Barthel Index and handgrip and knee extension strength significantly improved during hospitalization in iron deficiency and non-iron deficiency groups. Knee extension strength showed better improvement in iron-deficient patients receiving iron supplementation and iron supplementation during hospital stay was the main predictor for improvement in knee extension strength. Comorbidity, iron deficiency, and changes in handgrip strength were the major independent risk factors for poor improvement in Barthel Index during hospitalization. There were significant associations between patients’ fatigue and iron deficiency, comorbidity, and female gender.

**Conclusion:**

Iron deficiency is an independent risk factor for fatigue and poor functional recovery among older hospitalized patients. Iron supplementation seems to be capable of improving functional performance.

## Introduction

Iron deficiency is the most common nutritional deficiency worldwide, which includes absolute iron deficiency and functional iron deficiency [1, 2]. Absolute iron deficiency is defined as depleted body iron stores due to an imbalance between iron uptake and utilization [3, 4]. Low serum ferritin concentrations (<30 ng/l) are the hallmark of absolute iron deficiency [5, 6]. Functional iron deficiency arises due to reduced availability of iron stores even when storage iron is normal or increased [7]. Iron deficiency is a very common finding in older persons with prevalence rates from 11% among community-dwelling adults aged ≥65 years to over 50% in nursing home residents and in geriatric inpatients [8–10]. Anemia, in which iron deficiency is one of the major causes, is a well-known risk factor for impaired activities of daily living (ADL) in older persons [10]. Indeed, it is associated with a decline in functional capacity, fatigue, a higher rate of hospitalization, impaired cognitive function, depression, and increased risk of falling and mortality [10–12]. Findings of an observational study among 579 older hospitalized patients aged ≥70 years demonstrated a significant lower ADL, as measured by Barthel Index (BI), in anemic patients [10]. However, whether there is a causative relationship remains unclear.

Noteworthy, anemia in geriatric patients is usually a consequence of multimorbidity and rarely caused by one single reason [13]. However, poor nutritional intake of iron [3] and diseases accompanied by inflammation followed by reduced iron absorption and availability are risk factors associated with systemic iron depletion [3, 14], due to increased inflammatory hepcidin expression, a regulator of intestinal iron absorption and metabolic iron availability [12]. Iron acts as an oxygen-binding element and is therefore crucial for oxygen supply of the organism [15]. Iron is not only an essential component of hemoglobin (Hb) and erythropoiesis but also of myoglobin and mitochondrial enzymes. Accordingly, iron deficiency may have important effects on muscle function, oxidative energy metabolism, immune, and nervous system [15, 16].

Previous experimental animal studies revealed that iron deficiency, independent from anemia, causes functional impairments of skeletal muscle [17, 18]. In addition, a possible negative influence of iron deficiency on muscle function has been already reported in different populations. In a study of 44 chronic heart failure patients, Melenovsky et al. [19] found that heart failure patients with iron deficiency displayed severe myopathy compared those without iron deficiency. Furthermore, an impaired skeletal muscle function due to non-anemic iron deficiency was observed in chronic obstructive pulmonary disease patients that led to lower pretraining aerobic capacity and reduced training-induced response in these patients [20]. However, much of the evidence regarding the associations of iron deficiency and functional status were constricted to cross-sectional studies with no proof of causality. Longitudinal data of functional impairment in older hospitalized patients with iron deficiency are scarce.

Beyond the possible adverse effect of iron deficiency on muscle function, nonspecific symptoms such as fatigue and weakness are associated with iron deficiency [5, 21], therefore it may contribute to a reduced recovery. There are controversial results with respect to the association between iron deficiency and fatigue in several randomized controlled trials, cross-sectional and observational studies. In randomized controlled trails, Vauchere et al. [22] and Krayenbuehl et al. [23] demonstrated a significant therapeutic effect of iron supplementation on fatigue improvement in non-anemic iron deficiency patients whereas others did not find such an association [24, 25]. In a multicenter prospective study among 107 ICU patients aged 48–73 years, iron deficiency was associated with increased fatigue, independently of anemia [26]. In addition, meta-analyses indicated that improving iron status might be effective in decreasing fatigue in patients with non-anemic iron deficiency [27].

In this study, we sought to investigate whether iron deficiency is an independent risk factor for low skeletal muscle function, functional impairment, fatigue, and rehabilitation progress in older hospitalized patients.

## Subjects and methods

This prospective longitudinal observational study was undertaken between November 2018 and July 2019 at the geriatric department of the university hospital Marien Hospital Herne in Germany. The study participants comprise 224 consecutive older hospitalized patients with mean age 81.4 ± 6.2 years (67% females). Patient eligibility criteria were age ≥65 years, a probable stay of at least 14 days in hospital, ability to understand and cooperate and written informed consent. Subjects with known neuromuscular disease, paralysis of arm or leg, and no written informed consent were excluded. All patients had physical training for at least 30 min twice a day as a routine rehabilitation program. Furthermore, individualized training program was provided to all patients according to the deficient in activity of daily living. The attending physician recorded the clinical routine data by either interviewing the patients or asking their proxy. All research related data were obtained and recorded by the first author. Study data were collected and managed using REDCap electronic data capture tool [28] hosted at the university hospital Marien Hospital Herne. The study protocol had been approved by the ethical committee of Ruhr-University Bochum (number 18-6365 approved on 24.08.2018).

### Laboratory methods

Blood samples were obtained on admission or the following day and measured according to standard clinical procedure. Serum ferritin and transferrin were measured by electrochemiluminescence (ECLIA) and nephelometric methods, respectively. According to measured ferritin, iron, transferrin in serum, and blood Hb, patients were grouped into two categories as having iron deficiency or not. The percentage of transferrin saturation (TSAT) was calculated by using the formula; serum iron µg/dl × 70.9/serum transferrin mg/dl [29]. Low ferritin and low TSAT were defined as <30 ng/l and <16%, respectively [5]. The cut-off values of Hb (<13 mg/dl for men and <12 mg/dl for women) were taken from the guidelines of World Health Organization [30].

Patients with absolute iron deficiency were further classified as iron deficiency with anemia (serum ferritin <30 ng/l, TSAT <16%, Hb <13 mg/dl for men and <12 mg/dl for women) and iron deficiency without anemia (serum ferritin <30 ng/l, TSAT <16%, Hb ≥13 mg/dl for men and ≥12 mg/dl for women). In addition, functional iron deficiency was defined as serum ferritin ≥30 ng/l and TSAT <16%. Patients with serum ferritin ≥30 ng/l and TSAT ≥16% were considered as having no iron deficiency. Previous intake of iron supplementation was derived from the patients’ medical records or by interview. In addition, current iron supplementation during hospitalization was recorded. C-reactive protein (CRP) was measured on admission according to standard clinical procedures and a level greater than 3.0 (mg/dl) was defined as inflammation relevant for the development of functional iron deficiency [31].

### Geriatric assessment

All geriatric assessments were performed on admission except the BI, which was evaluated on admission and at the time of discharge. BI was used to assess self-caring activities [32] and a positive difference between BI on admission and discharge was considered as an indicator of rehabilitation success. The point’s range of the German version of the BI is 0–100 pts., with 100 pts. indicating independence in all ADL. Frailty was diagnosed based on the FRAIL scale [33] with score 0 being not frail, 1–2 prefrail, and 3–5 frail. The risk of sarcopenia was evaluated based on SARC-F questionnaire [34, 35] with a total score of 10 and subjects with score ≥4 were defined as having probable sarcopenia. Charlson Comorbidity Index [36] was used to determine medical comorbidities. Fatigue and fatigue severity was measured using the fatigue severity scale which covers nine items that can be scored from 1 (strong disagreement) to 7 (strong agreement) and patients with a mean score ≥5 were considered as having significant fatigue [37]. Physical performance was measured with short physical performance battery (SPPB) [38] with 8 points or lower indicating impaired physical performance.

A Jamar dynamometer (Lafayette Instrument Company, Lafayette, IN) was used to assess handgrip strength and the protocol described by Gandevia [39] was performed to measure isometric knee extension strength on admission and at time of discharge. For knee extension strength, patient was seated on a chair with a strap around his leg 10 cm above the ankle joint whereas his hip and knee joint angles positioned at 90 degrees. Handgrip and knee extension strength were assessed three times at the dominant or unaffected side of hand/leg and the maximum score was recorded.

### Data analysis

All statistical analysis was performed using SPSS statistical software (SPSS Statistics for Windows, IBM Corp, Version 25.0, Armonk, NY, USA). According to previous studies, 10% difference in muscle strength and mean values (SD) of handgrip strength of 22 kg (±7 kg) in the group without iron deficiency would be considered clinically relevant. Therefore, with these expectations, a case number of *N* = 200 in 2:1 design with a power of 0.8 and a Type I error of 0.05 is calculated (www.clinical-trials.de). Means and standard deviations (SDs) were used for continuous data with normal distribution whereas median values are expressed with interquartile ranges for non-normally distributed data. Categorical variables are reported as absolute numbers and percentages (*n*, %). Differences between variables and between baseline and follow-up within each group (iron deficiency and non-iron deficiency groups) were analyzed by using paired samples *t*-test for normally distributed values. Differences in functional status at baseline and in magnitude of changes at follow-up between groups were analyzed by using an unpaired *t-*test in normally distributed variables and the Chi square test for categorical variables. A series of stepwise multiple regression analysis was performed to determine the impact of risk factors (i.e., diseases, iron deficiency, iron supplementation, age, gender, inflammation, handgrip, and knee extension strength as independent variables) on BI, SPPB, and fatigue on admission and changes in BI, changes in handgrip, and knee extension strength (as dependent variables). Statistical significance was set at *p* < 0.05.

## Results

Baseline characteristics and laboratory data of study participants stratified by iron status are summarized in Table 1. Major diagnoses defined as reason for hospital admission were cardiovascular diseases, falls, fractures, and primary neurodegenerative diseases. Of 224 patients, age range 65–95 years, 91 (41%) had iron deficiency in which the majority had functional iron deficiency (78/91, 86%). Absolute iron deficiency with and without anemia was diagnosed in 12 (13%) and 1 (1%) patients, respectively. In addition, functional iron deficiency with and without anemia was seen in 65 (72%) and 13 (14%) patients, respectively. Fifty nine percent of total population displayed no iron deficiency.

**Table 1:** Characteristic of study population at baseline stratified by iron status.

	All (*n* = 224)	Iron deficiency (*n* = 91; 41%)	No iron deficiency (*n* = 133; 59%)
Gender (*n*,%)			
Male	75 (33.0%)	37 (41.0%)	38 (29.0%)
Female	149 (67.0%)	54 (59.0%)	95 (71.0%)
Age (year)	81.4 ± 6.2	80.4 ± 6.9	82.12 ± 5.7
Height (m)	1.66 ± 8.4	1.66 ± 8.0	1.66 ± 8.2
Actual body weight (kg)	73.0 ± 16.4	72.3 ± 16.6	73.5 ± 16.3
BMI (kg/m^2^)	26.4 ± 5.6	26.0 ± 5.8	26.6 ± 5.2
Barthel Index on admission, median (IQR)	50 (45–60)	45 (45–55)	50 (45–62.5)
Frail simple scale score, median (IQR)	4 (3–4)	4 (3–4)	3.5 (2–4)*
SARC‐F scores, median (IQR)	6 (4–8)	6 (4–8)	6 (4–8)
Fatigue severity scale score, median (IQR)	4.6 (3–5.7)	5.2 (3.3–6.1)	4.2 (2.8–5.3)**
SPPB, median (IQR)	3 (2–5)	4 (2–5)	3 (2–5)
CCI score, median (IQR)	3 (1–5)	3 (2–6)	3 (1–4)
Iron supplementation before admission			
Yes (*n*,%)	55 (25.0%)	28 (31.0%)	27 (20.0%)*
No (*n*,%)	169 (75.0%)	63 (69.0%)	106 (80.0%)
Iron supplementation during hospitalization			
Yes (*n*,%)	51 (23.0%)	41 (45.0%)	10 (7.0%)***
No (*n*,%)	173 (77.0%)	50 (55.0%)	123 (93.0%)
Number of drugs/day, median (IQR)	9 (6–11)	9 (7–11)	9 (6–11)
CRP (mg/dl)	2.4 ± 3.8	3.2 ± 5.1	1.9 ± 2.5**
Ferritin (ng/ml)	260.7 ± 290.3	224.0 ± 295.7	285.7 ± 285.0
TSAT (%)	20.8 ± 3.8	11.0 ± 3.2	27.6 ± 11.9***
Hb (g/dl)			
Male	11.8 ± 2.1	11.1 ± 1.9	12.4 ± 2.1**
Female	11.3 ± 1.7	10.7 ± 1.5	11.7 ± 1.7***
Length of hospital stay	16 (14–21)	14 (14–21)	18 (14–20)

Values are given as mean ± SD, number (%), or median (IQR, interquartile range).

*SPPB* short physical performance battery, *CCI* Charlson Comorbidity Index, *CRP* C-reactive protein, *TSAT* transferrin saturation, *Hb* hemoglobin.

*p < 0.05; **p < 0.01; ***p < 0.001, difference between groups.

Frailty and fatigue scores were significantly higher in those with iron deficiency. Compared with the non-iron deficiency group, patients with iron deficiency had lower Hb and higher CRP levels. The median length of hospital stay was 16 days. Almost half of the patients with iron deficiency received iron supplementation during hospital stay in whom severity of iron deficiency was higher compared to the patients not receiving supplements (mean Hb 10.4 ± 1.6 g/dl vs. 11.1 ± 1.7 g/dl, *p* = 0.044; mean TSAT 10.5 ± 3.4% vs. 11.5 ± 3.0%, *p* = 0.599, respectively). Depending on the clinical situation of patients and the attending physician judgment, the majority of patients (49/51; 96%) received oral iron supplementation (100 mg/daily) and only two patients (4%) received intravenous iron during hospital stay.

Muscle strength and functional status on admission and the respective changes during hospitalization stratified by iron status are presented in Table 2. There were no significant differences in handgrip strength, knee extension strength, total BI score, and the respective BI item “walking” between iron deficiency and non-iron deficiency groups on admission and at discharge. However, the median score of the respective BI item “climbing stair” was significantly higher in non-iron deficiency group on admission (*p* = 0.032).

**Table 2:** Changes in functional status during hospitalization in total population stratified by iron status.

	Iron deficiency (*n* = 91; 41%)		No iron deficiency (*n* = 133; 59%)	
Total (*n* = 224)	Baseline	Follow-up	Baseline	Follow-up
Handgrip strength (kg)	20.6 ± 9.4	21.9 ± 9.7*	20.3 ± 7.6	21.4 ± 7.5***
Isometric knee extension strength (kg)	13.8 ± 6.4	16.4 ± 7.4***	13.7 ± 6.2	15.4 ± 6.5***
Total Barthel Index (median, IQR)	45 (45–55)	75 (65–85)***	50 (45–62.5)	75 (63.7–81.2)***
Walking	5 (5–10)	10 (10–10)***	5 (5–10)	10 (10–10)***
Climbing stairs	0 (0-0)	5 (0–5)***	0 (0–5)^†^	5 (0–5)***

*p < 0.05; ***p < 0.001, difference within groups; ^†^*p* < 0.05, difference between groups at baseline.

Significant improvement was observed in handgrip strength and isometric knee extension strength over time in iron deficiency and non-iron deficiency groups (+1.3 kg vs. +1.1 kg for handgrip strength, *p* = 0.380 and +2.6 kg vs. +1.7 kg for knee extension strength, *p* = 0.125, respectively). Furthermore, total BI score and its respective items “walking and climbing stairs” substantially increased during hospitalization in both groups with no significant differences between groups (+30 and +25 median change in BI in iron and non-iron deficiency groups, respectively; *p* = 0.173). In addition, significant positive associations were observed between Hb levels and handgrip strength on admission (*p* = 0.049) and at discharge (*p* = 0.004) in iron deficiency group only. In this group, those with higher levels on admission had a marked improvement in handgrip strength during hospitalization compared to those with lower Hb levels (+2.5 kg vs. 0.5 kg, *p* = 0.035, respectively).

Table 3 shows the association of iron supplementation with changes of functional status during hospital stay in the sub-group of patients with iron deficiency. Patients with iron deficiency who received supplementation during hospital stay had significantly higher knee extension strength on admission and at the time of discharge compared to those not receiving supplementation. It is worth mentioning that 57% of iron-deficient patients who received iron supplementation before admission continued with supplementation during hospital stay in whom knee extension strength was significantly higher compared to those receiving supplementation neither before admission nor during hospital stay (15.5 kg vs. 12.9 kg, *p* = 0.005, respectively).

**Table 3:** Impact of iron supplementation on functional status during hospitalization in a sub-group of patients with iron deficiency.

	Iron deficiency (*n* = 91)		
	Iron supplementation (*n* = 41)	No iron supplementation (*n* = 50)	*p* value*
On admission			
Handgrip strength (kg)	22.0 ± 9.2	20.0 ± 9.5	0.203
Isometric knee extension strength (kg)	15.9 ± 6.9	12.1 ± 5.6	0.008
Total Barthel Index (median, IQR)	50 (45–55)	45 (45–55)	0.878
Walking	5 (5–10)	5 (5–10)	0.771
Climbing stairs	0 (0–0)	0 (0–0)	0.756
At discharge			
Handgrip strength (kg)	23.0 ± 9.4	21.0 ± 9.9	0.734
Isometric knee extension strength (kg)	19.1 ± 7.8^†^	14.3 ± 6.4^†^	0.005
Total Barthel Index (median, IQR)	70 (60–80)^†^	75 (67–85)^†^	0.118
Walking	10 (10–10)^†^	10 (10–10)^†^	0.827
Climbing stairs	5 (0–5)^†^	5 (5–5)^†^	0.072

*Difference between groups; ^†^*p* < 0.001, difference within group from admission to discharge.

In addition, knee extension strength significantly increased in both groups. Most importantly, the improvement of knee extension strength was better in iron-deficient patients receiving iron supplementation compared to those not receiving supplementation (23% vs. 16%, respectively; Table 3), although it did not reach the level of significance (*p* = 0.076).

To test the independent effects of factors such as age, gender, inflammation, comorbidity, iron deficiency, iron supplementation (previous intake or during hospital stay), and handgrip and knee extension strength on fatigue, SPPB, and total BI score on admission (as dependent variables), we performed a series of stepwise multiple regression analysis (Table 4). In addition, the effects of aforementioned factors on changes in strength measurements and total BI score (as dependent variables) were tested. Iron deficiency, comorbidity, and female gender were the major independent risk factors for patients’ fatigue on admission accounting for 28.5% of the variance. Iron deficiency, gender (female), and handgrip strength entered the prediction model accounting for 38.8% of the variance in SPPB on admission, whereas iron deficiency and handgrip strength accounted for 24.0% of the variance in BI on admission (Table 4).

**Table 4:** Stepwise multiple regression analysis of risk factors associated with fatigue and functional status on admission and during hospital stay in total population.

	Beta coefficient	SE	*p* value
Fatigue			
Charlson Comorbidity Index	0.104	0.042	0.014
Iron deficiency or not	−0.534	0.227	0.019
Previous iron supplementation or not	0.061	0.192	0.971
Age	−0.074	0.237	0.269
Gender^a^	0.768	0.239	<0.001
C-reactive protein	0.093	0.264	0.173
SPPB on admission			
Charlson Comorbidity Index	0.030	0.453	0.651
Iron deficiency or not	−0.529	0.268	0.049
Previous iron supplementation or not	−0.063	0.789	0.331
Age	−0.067	0.018	0.297
Gender^a^	1.405	0.324	<0.001
C-reactive protein	−0.007	0.863	0.916
Handgrip strength	0.104	0.018	<0.001
Barthel Index on admission			
Charlson Comorbidity Index	−0.031	0.455	0.649
Iron deficiency or not	3.913	1.797	0.031
Previous iron supplementation or not	−0.097	0.416	0.158
Age	−0.068	0.992	0.322
Gender	0.152	0.916	0.057
C-reactive protein	−0.107	0.575	0.117
Handgrip strength	0.242	0.116	0.039
knee extension strength	0.065	0.817	0.415
Changes in Barthel Index			
Charlson Comorbidity Index	−0.872	0.336	0.010
Iron deficiency or not	−3.917	1.830	0.034
Previous iron supplementation or not	0.045	0.657	0.512
Actual iron supplementation or not	−0.042	0.552	0.581
Age	0.005	0.071	0.944
Gender	0.113	0.594	0.113
C-reactive protein	−0.109	0.565	0.119
Changes in handgrip strength	0.641	0.274	0.020
Changes in knee extension strength	0.099	0.402	0.163
Changes in knee extension strength			
Charlson Comorbidity Index	−0.065	0.925	0.356
Iron deficiency or not	−0.052	0.680	0.498
Previous iron supplementation or not	−0.108	0.514	0.132
Actual iron supplementation or not	1.150	0.495	0.021
Age	0.047	0.668	0.505
Gender	0.124	0.730	0.085
C-reactive protein	0.116	0.657	0.099

*SE* standard error, *SPPB* short physical performance battery.

^a^Female gender.

Furthermore, comorbidity, iron deficiency, and changes in handgrip strength were the major independent risk factors for changes in BI during hospital stay accounting for 27.0% of the variance. Iron supplementation during hospital stay was the main predictor for improvement in knee extension strength accounting for 16.0% of the variance. None of the variables contributed significantly to the variance of changes in handgrip strength. Moreover, significant associations between gender and comorbidity (*p* = 0.001), handgrip (*p* < 0.001), and knee extension strength (*p* = 0.001) on admission, between BI and SBBP (*p* < 0.001) and handgrip strength (*p* = 0.035) on admission and between changes in BI and changes in handgrip (*p* = 0.018) and knee extension strength (*p* = 0.032) were observed.

## Discussion

The findings of the present study indicated that 41% of older hospitalized patients had iron deficiency in which the majority (86%) suffered from functional iron deficiency. Iron deficiency is relatively common and frequently diagnosed in older individuals. In a hospital-based prospective observational study among 105 older patients, iron deficiency was found in 26 (24.8%) patients [40]. In addition, results of a cross-sectional, retrospective analysis of inpatients and outpatients aged ≥64 years demonstrated that 21.1% of the patients were anemic in which 14.4% and 28.2% had absolute and functional iron deficiency, respectively [41].

In this study, low SPPB, BI, and fatigue on admission were found to be significantly associated with iron deficiency. Moreover, iron deficiency followed by comorbidity was the major independent risk factors for changes in BI during hospital stay in our population. In line with our results, in a multicenter study among 109 outpatients with heart failure (mean age 71 ± 9 years) Bekfani et al. [42] reported a worse exercise capacity in 6-min walking test and lower muscle strength and quality-of-life in patients with iron deficiency compared to those without iron deficiency. Findings of another study among 138 chronic systolic heart failure patients (mean age 60.9 years) indicated a significant association between absolute iron deficiency without anemia and poorer functional capacity [43]. All these data suggest that beyond physiological muscle atrophy with advancing age, iron deficiency may contribute to impairment of muscle function and muscle strength of older persons. It should be noted that in this study, BI and muscle strength improved during hospital stay in both iron-deficient and non-iron-deficient groups. This improvement is supposed to be the effect of natural recovery from disease and the routine rehabilitation program in our geriatric acute care unit, i.e., physical and occupational training in all patients.

Another important finding of our study is that iron deficiency was an independent risk factor for patients’ fatigue. Fatigue is a common condition among older individuals with a prevalence range of 29% at age 70 years to 68% at age 85 years [44]. Significant positive associations between fatigue and iron deficiency have been reported in previous studies [26, 27]. Results of a multicenter prospective study among ICU patients aged 48–73 years demonstrated a significant link between iron deficiency and fatigue, independently from anemia [26]. In a systematic review and meta-analysis of randomized controlled trials, Houston et al. [45] showed a significant effect of iron supplementation on reduction of subjective measures of fatigue among iron-deficient non-anemic individuals. Beyond the fact that iron deficiency is a major cause of anemia, it is also associated with fatigue and poor functional status and may thus impair/or reduce patient’s recovery. Indeed, iron deficiency may act through fatigue leading to a lower functional performance. On the other hand, older individuals with iron deficiency may be less active, which results in muscular weakness and decline of muscle strength due to disuse. Therefore, all of these factors could explain a greater physical dysfunction in older patients with iron deficiency.

It is worth mentioning that in this study, severity of iron deficiency was higher in iron-deficient patients who received iron supplementation compared to the patients not receiving supplements. Despite the fact that some of the iron-deficient patients did not receive iron supplementation, it may be speculated that the severity of iron deficiency was a trigger for iron supplementation in this observational study. We found that patients with iron deficiency who received iron supplementation during hospital stay and before admission had significantly higher knee extension strength at baseline and follow up, compared to those not receiving any supplements. In addition, functional status was improved over time in both groups. However, we observed an obvious tendency toward a higher improvement in isometric knee extension strength during hospital stay in supplemented group, although it did not reach the level of significance likely due to the small number of patients and the short duration of iron supplementation. Our findings suggest that iron supplementation may influence physical performance and therefore affects geriatric rehabilitation. Accordingly, findings of a randomized, double-blind study among 459 patients with chronic heart failure with iron deficiency (mean age 67.8 years) demonstrated a significant improvement in the distance on the 6-min walk test and quality-of-life with intravenous iron supplementation (ferric carboxymaltose) [46].

This study has some limitations. Due to nonspecific utilization of plasma transferrin receptor concentration in older individuals, TSAT was used as an indicator of iron deficiency in this study. We grouped the patients into two categories as having iron deficiency (all forms) or not due to small number of patients in the subgroups of iron deficiency. Since the majority of iron-deficient patients had functional iron deficiency and very few patients displayed absolute iron deficiency with and without anemia, we were unable to further investigate the differences in functional changes within the subgroups of iron deficiency. In addition, data regarding iron supplementation before admission were derived either from the patients’ medical records or by interview. Therefore, it cannot be excluded that some patients had previous iron supplementation without respective information in the record. Further prospective interventional research is required to determine whether the treatment of iron deficiency (i.e., using iron supplementation) results in preservation or improvement of physical function and muscle strength of older adults.

## Conclusion

The results of this study indicate that iron deficiency is an independent risk factor for fatigue and poor functional recovery among older hospitalized patients. Accordingly, iron supplementation seems to be capable of improving functional performance in iron-deficient older subjects.
